# Supplementary material for: Synergistic Effects of Metformin and Trastuzumab on HER2 Positive Gastroesophageal Adenocarcinoma Cells In Vitro and In Vivo
Source: Cancers (Basel). 2023 Sep 28;15(19):4768. doi: 10.3390/cancers15194768 (PMC10571931; doi:10.3390/cancers15194768)
Supplement: Supplementary file 1 [file cancers-15-04768-s001.zip › Supplementary Figure S1.pptx]

## Slide 1
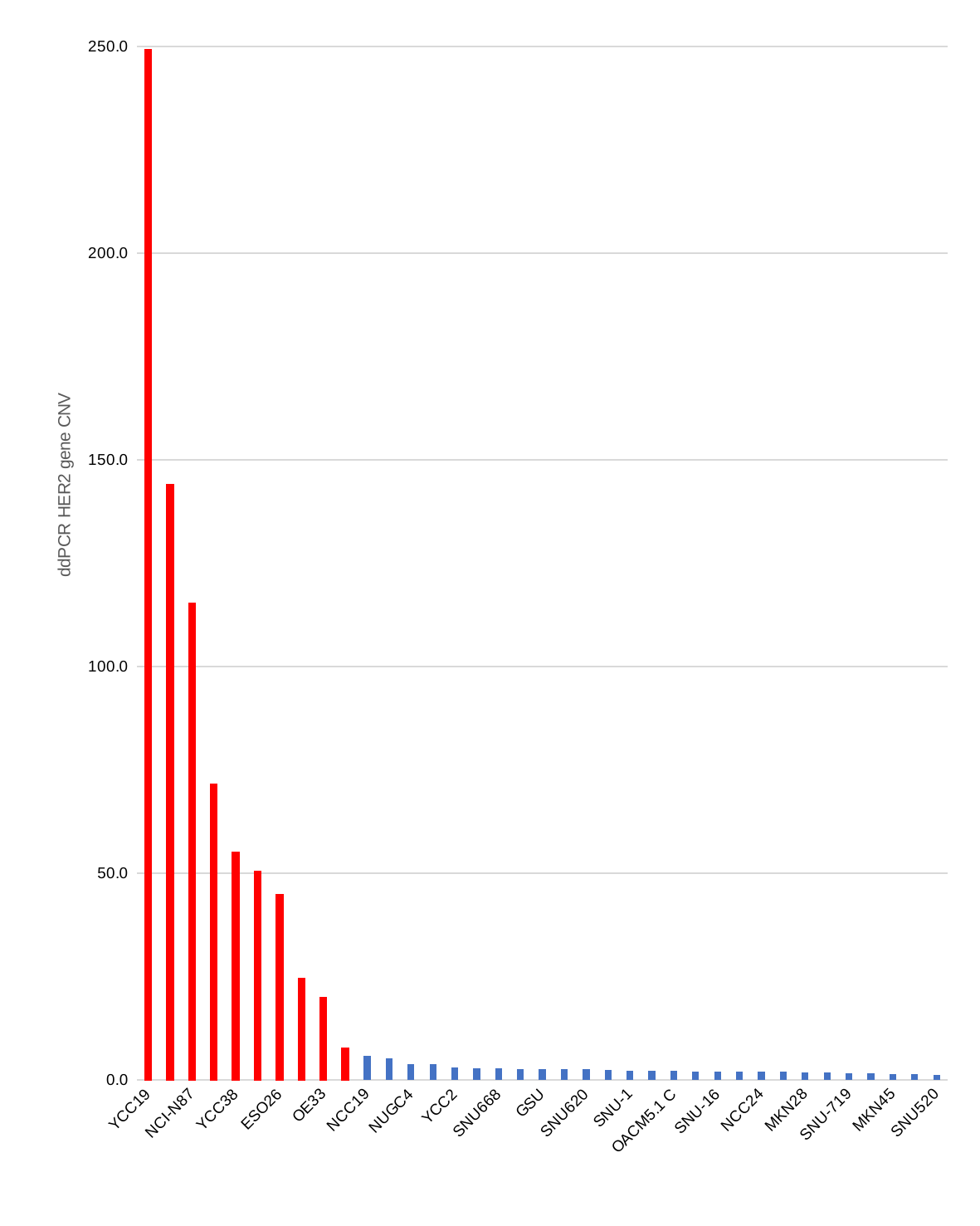

### Chart: ddPCR HER2 gene CNV
| Category | |
|---|---|
| YCC19 | 249.28787224859732 |
| OE19 | 144.0 |
| NCI-N87 | 115.4 |
| YCC33 | 71.60928742994396 |
| YCC38 | 55.232520325203254 |
| KYAE1 | 50.5 |
| ESO26 | 44.967320261437905 |
| MKN7 | 24.51502145922747 |
| OE33 | 20.0 |
| SNU216 | 7.683304647160069 |
| NCC19 | 5.884941842187991 |
| YCC7 | 5.155855096882898 |
| NUGC4 | 3.9 |
| YCC3 | 3.880409784653983 |
| YCC2 | 3.094979352314714 |
| ESO51 | 2.9 |
| SNU668 | 2.7883482067688163 |
| SNU638 | 2.7 |
| GSU | 2.69811320754717 |
| KATO-Ⅲ | 2.6027397260273974 |
| SNU620 | 2.6 |
| SNU1750 | 2.484076433121019 |
| SNU-1 | 2.2652043868394816 |
| YCC42 | 2.218978102189781 |
| OACM5.1 C | 2.2 |
| SNU-601 | 2.0172091283202396 |
| SNU-16 | 2.0012202562538133 |
| AGS | 2.0008133387555915 |
| NCC24 | 2.0 |
| SNU5 | 2.0 |
| MKN28 | 1.7367565810854728 |
| SNU-1967 | 1.7328800388538126 |
| SNU-719 | 1.71125662613034 |
| SNU-484 | 1.539622641509434 |
| MKN45 | 1.5 |
| NCC59 | 1.4778625954198474 |
| SNU520 | 1.3 |
