# Supplementary material for: Synergistic Effects of Metformin and Trastuzumab on HER2 Positive Gastroesophageal Adenocarcinoma Cells In Vitro and In Vivo
Source: Cancers (Basel). 2023 Sep 28;15(19):4768. doi: 10.3390/cancers15194768 (PMC10571931; doi:10.3390/cancers15194768)
Supplement: Supplementary file 1 [file cancers-15-04768-s001.zip › Supplementary Table S1. Cell line list.pdf]

Supplementary Table S1. Gastroesophageal cancer cell lines

	Cell lines	Description	Vendor
1	NCI-N87	Gastric adenocarcinoma	Korean Cell Line Bank
2	MKN-28	Gastric adenocarcinoma	Korean Cell Line Bank
3	SNU-216	Gastric adenocarcinoma	Korean Cell Line Bank
4	NCC-19	Gastric adenocarcinoma	Korean Cell Line Bank
5	NCC-24	Gastric adenocarcinoma	Korean Cell Line Bank
6	NCC-59	Gastric adenocarcinoma	Korean Cell Line Bank
7	SNU-520	Gastric adenocarcinoma	Korean Cell Line Bank
8	SNU-1967	Gastric adenocarcinoma	Korean Cell Line Bank
9	SNU-1750	Gastric adenocarcinoma	Korean Cell Line Bank
10	GSU	Gastric adenocarcinoma	Riken BioResource Research Center
11	NUGC-4	Gastric adenocarcinoma	Riken BioResource Research Center
12	MKN-7	Gastric adenocarcinoma	Riken BioResource Research Center
13	OE19	Oesophageal adenocarcinoma	Sigma-Aldrich
14	OE33	Oesophageal adenocarcinoma	Sigma-Aldrich
15	ESO26	Adenocarcinoma of the gastroesophageal junction	Sigma-Aldrich
16	ESO51	Oesophageal adenocarcinoma	Sigma-Aldrich
17	KYAE-1	Oesophageal adenocarcinoma	Sigma-Aldrich
18	OACM5.1C	Oesophageal adenocarcinoma	Sigma-Aldrich
19	AGS	Gastric adenocarcinoma	Korean Cell Line Bank
20	KATO-Ⅲ	Gastric adenocarcinoma	Korean Cell Line Bank
21	MKN-45	Gastric adenocarcinoma	Korean Cell Line Bank
22	SNU-1	Gastric adenocarcinoma	Korean Cell Line Bank
23	SNU-5	Gastric adenocarcinoma	Korean Cell Line Bank
24	SNU-16	Gastric adenocarcinoma	Korean Cell Line Bank
25	SNU-484	Gastric adenocarcinoma	Korean Cell Line Bank
26	SNU-601	Gastric adenocarcinoma	Korean Cell Line Bank
27	SNU-620	Gastric adenocarcinoma	Korean Cell Line Bank
28	SNU-638	Gastric adenocarcinoma	Korean Cell Line Bank
29	SNU-668	Gastric adenocarcinoma	Korean Cell Line Bank
30	SNU-719	Gastric adenocarcinoma	Korean Cell Line Bank
31	YCC-2	Gastric adenocarcinoma	donated by Professor Sun Young Rha, Yonsei Cancer Center, Yonsei University, Seoul
32	YCC-3	Gastric adenocarcinoma	donated by Professor Sun Young Rha, Yonsei Cancer Center, Yonsei University, Seoul
33	YCC-7	Gastric adenocarcinoma	donated by Professor Sun Young Rha, Yonsei Cancer Center, Yonsei University, Seoul
34	YCC-19	Gastric adenocarcinoma	donated by Professor Sun Young Rha, Yonsei Cancer Center, Yonsei University, Seoul
35	YCC-33	Gastric adenocarcinoma	donated by Professor Sun Young Rha, Yonsei Cancer Center, Yonsei University, Seoul
36	YCC-38	Gastric adenocarcinoma	donated by Professor Sun Young Rha, Yonsei Cancer Center, Yonsei University, Seoul
37	YCC-42	Gastric adenocarcinoma	donated by Professor Sun Young Rha, Yonsei Cancer Center, Yonsei University, Seoul
