# Supplementary material for: Synergistic Effects of Metformin and Trastuzumab on HER2 Positive Gastroesophageal Adenocarcinoma Cells In Vitro and In Vivo
Source: Cancers (Basel). 2023 Sep 28;15(19):4768. doi: 10.3390/cancers15194768 (PMC10571931; doi:10.3390/cancers15194768)
Supplement: Supplementary file 1 [file cancers-15-04768-s001.zip › Supplementary Table S2. Primer sequence.pdf]

Supplementary Table S2. Primer sequence for droplet digital PCR

Gene	Primer Sequence	Probe Sequence
HER2	Forward, GGGGAGAATGTGAAAATTCCA Reverse, AGAGGGTGGAGGGGCTTA	CCAAAGCCAACAAAGAAATCT
PPIA (Reference 1)	Forward, ATTTTAGGATGTTTTTCGCATCTG Reverse, TGGCAGAATTTGTTCCTATGC	GGAATTGTCAAACTGACTCCTTTTCC
RPP30 (Reference 2)	Forward, GTTGCCAGTCATAGTGATTGTTC Reverse, GGCCTCCACAATATTCATGC	TTGGATGGCAAGCATGTGGT
AP3B1 type 1 (Reference 3)	Forward, AAGTGGAGAACAAGGCGAAA Reverse, CCGTCCACTCTCACTGTCCT	CAGCAGTGAGGACTCCTCCA
BT (Reference 4)	Forward, CAGTGATCCGCTTTAACAATCCT Reverse, GTCTGCATCTCACCGGCTTAA	GACACAGCAGCTGATGGAAA
