# Supplementary figures and images for: Synergistic Effects of Metformin and Trastuzumab on HER2 Positive Gastroesophageal Adenocarcinoma Cells In Vitro and In Vivo
Source: Cancers (Basel). 2023 Sep 28;15(19):4768. doi: 10.3390/cancers15194768 (PMC10571931; doi:10.3390/cancers15194768)

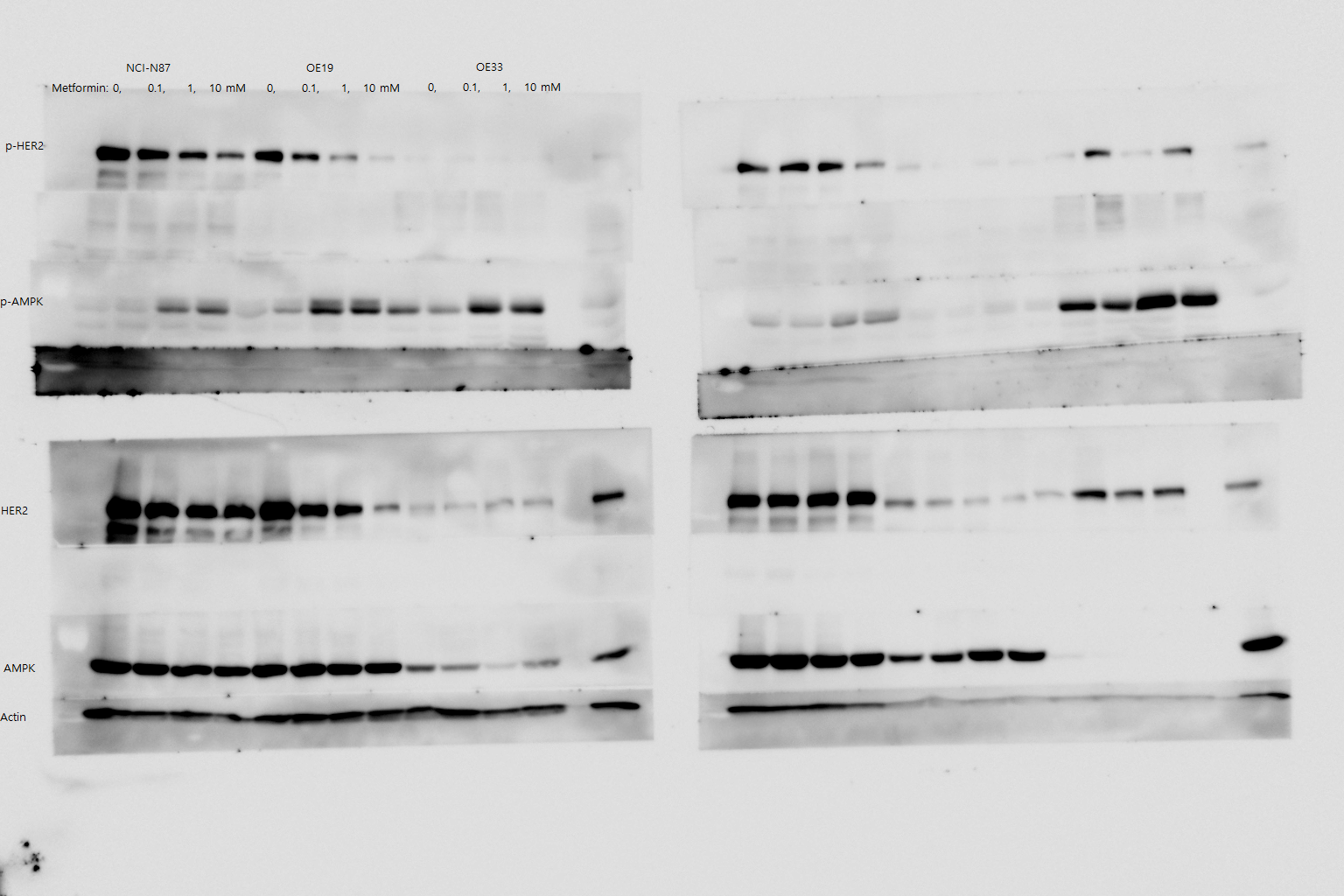

Supplement: Supplementary file 1 [file cancers-15-04768-s001.zip › cancers-2606105-File S1/Fig.1C/20170314_1127_7_p-HER2_p-AMPK_metformin titer_label.tif]

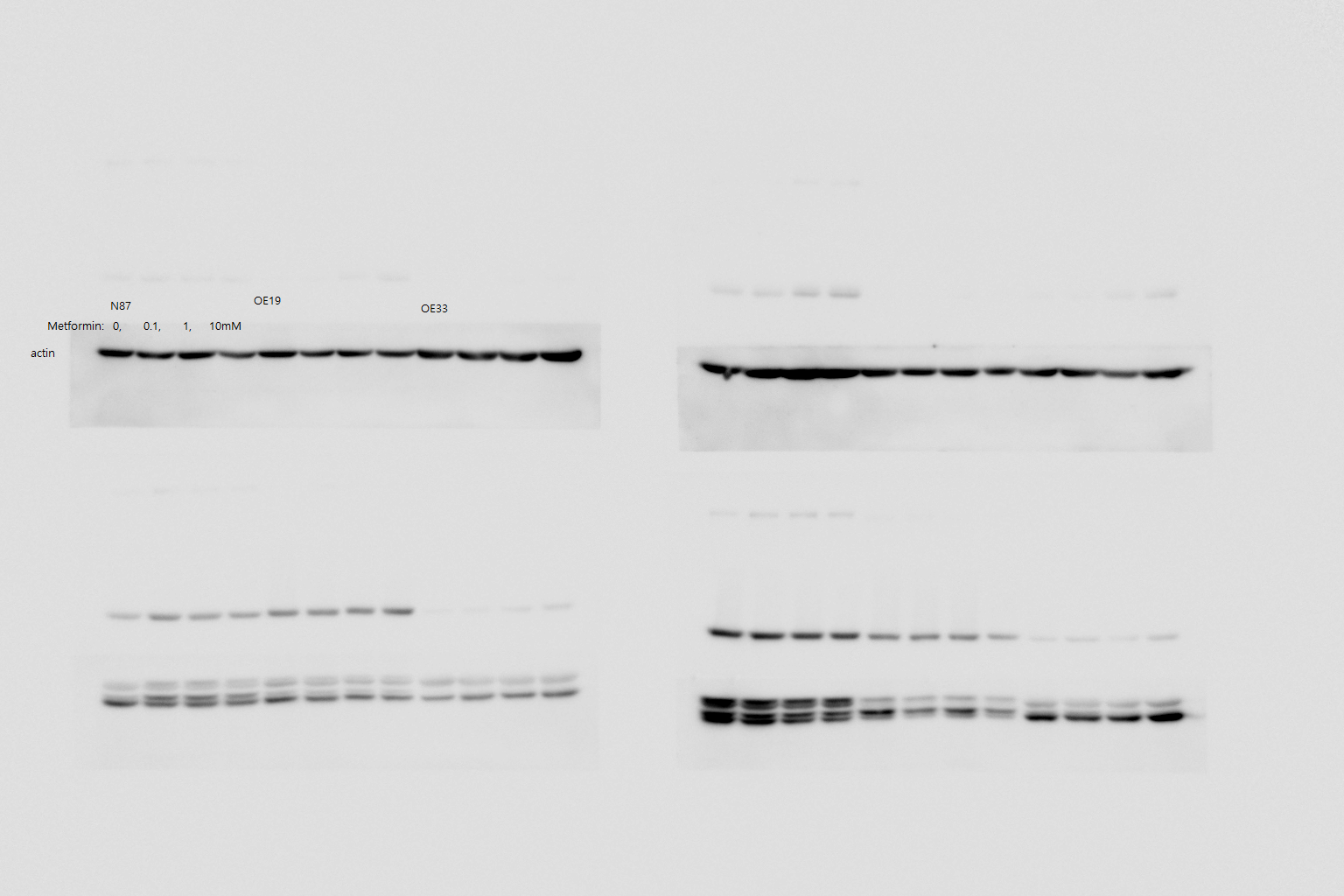

Supplement: Supplementary file 1 [file cancers-15-04768-s001.zip › cancers-2606105-File S1/Fig.1C/20170322_actin_metformin titer_label.tif]

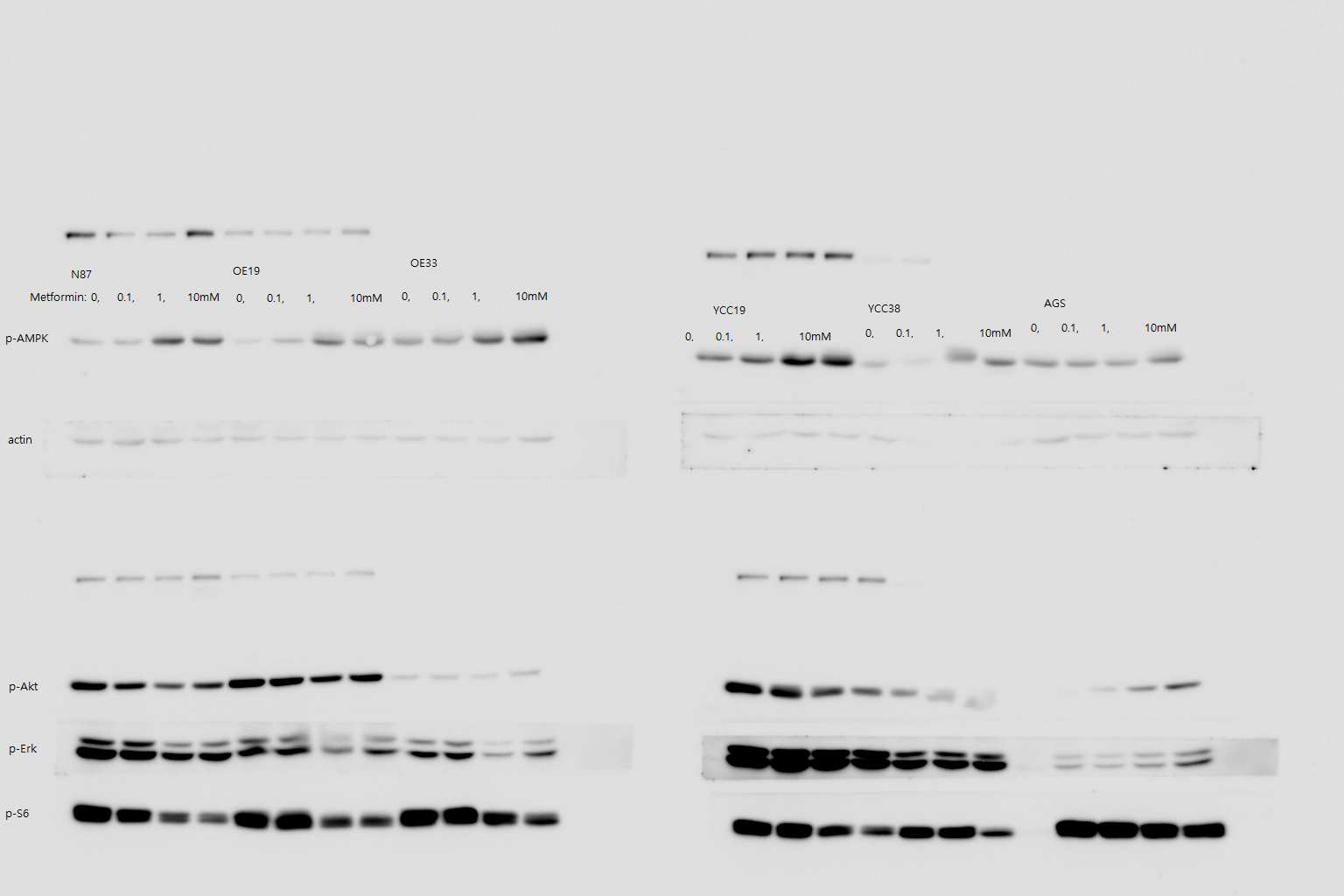

Supplement: Supplementary file 1 [file cancers-15-04768-s001.zip › cancers-2606105-File S1/Fig.1C/20170511_1720_4__p-AMPK_metformin titer_label.tif]

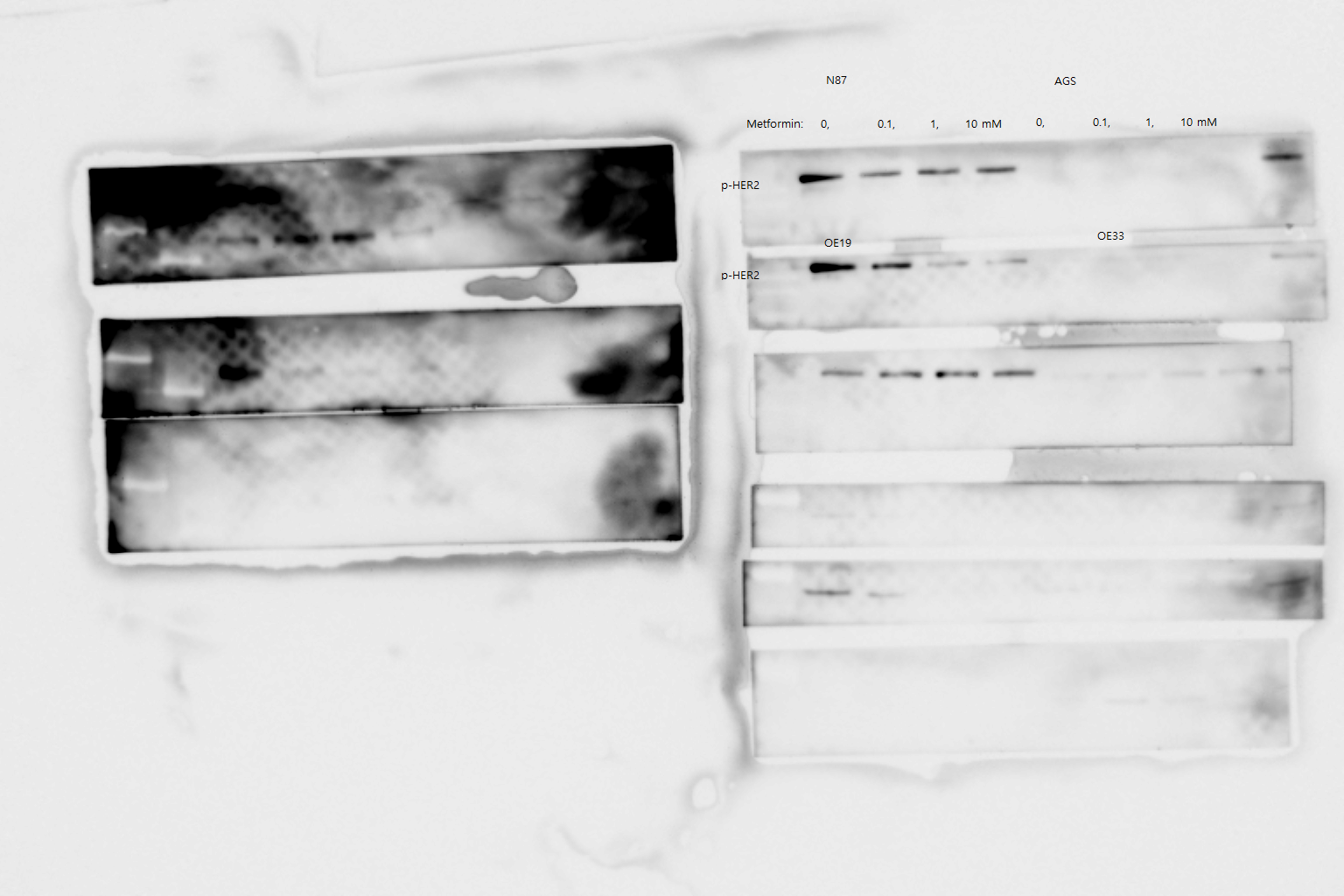

Supplement: Supplementary file 1 [file cancers-15-04768-s001.zip › cancers-2606105-File S1/Fig.1C/20171020_1136_7_p-HER2_metformin titer_label.tif]

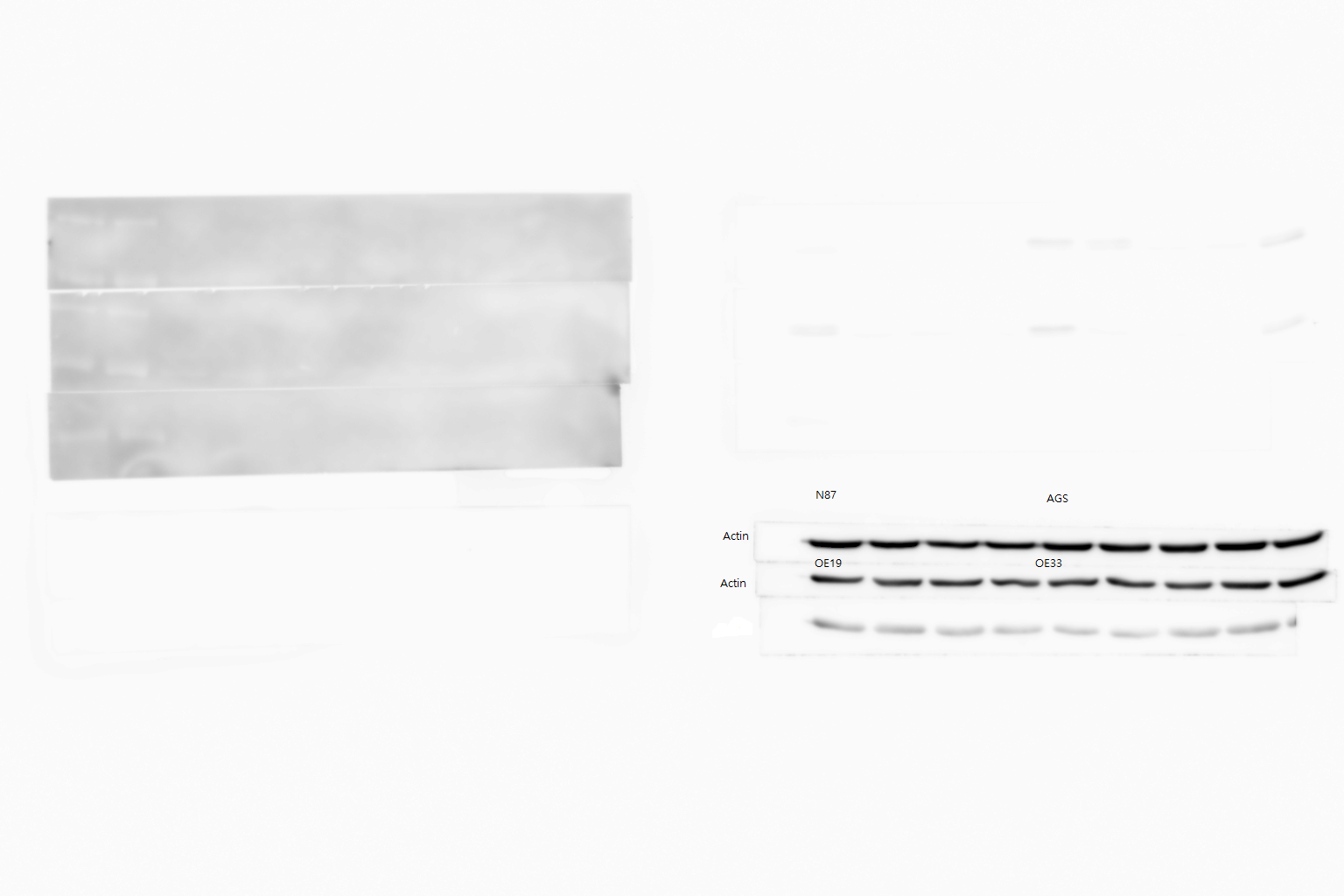

Supplement: Supplementary file 1 [file cancers-15-04768-s001.zip › cancers-2606105-File S1/Fig.1C/20171020_1145_3_actin_metformin titer_label.tif]

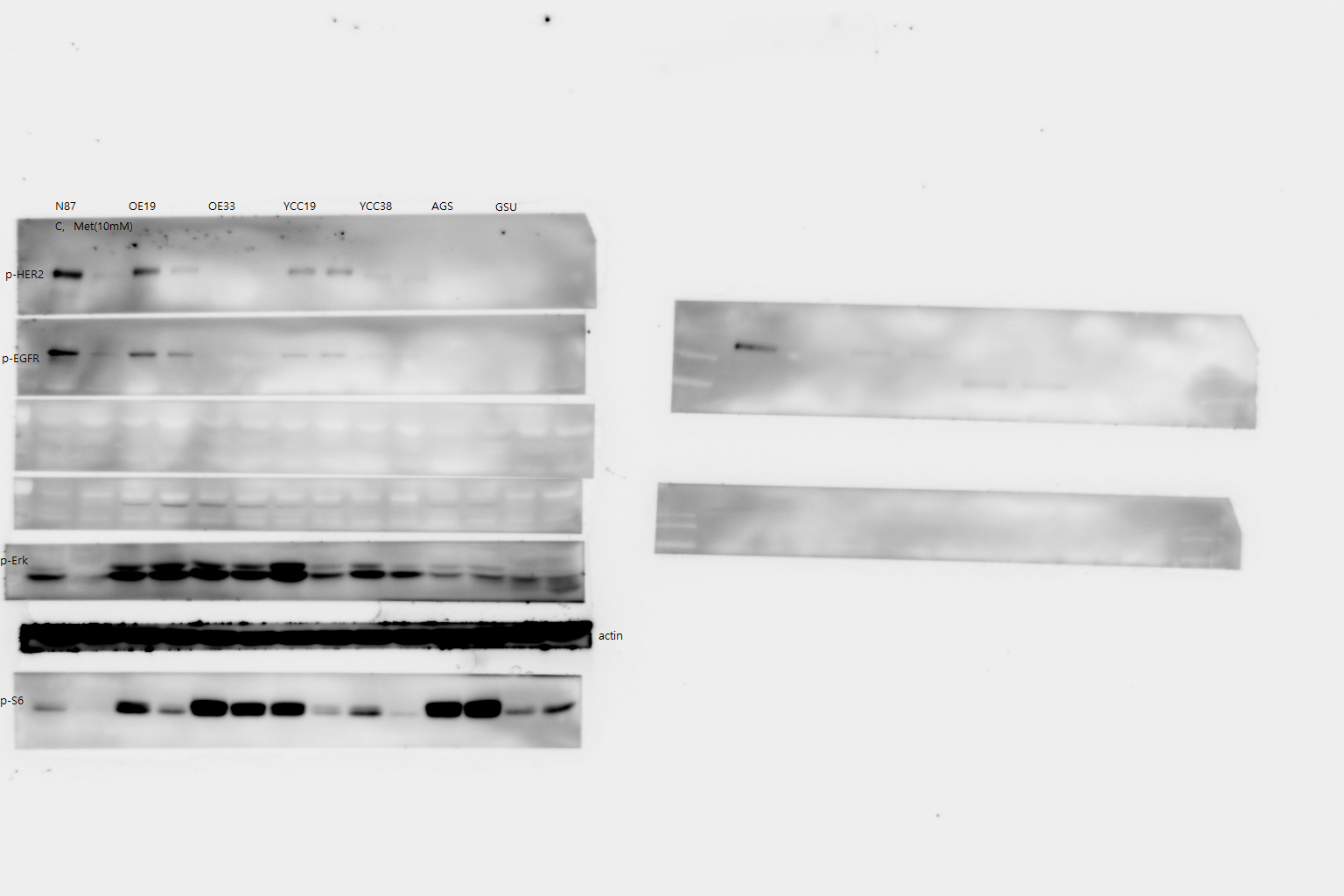

Supplement: Supplementary file 1 [file cancers-15-04768-s001.zip › cancers-2606105-File S1/Fig.2/20180531_1743_6_p-HER2_p-EGFR_label.tif]

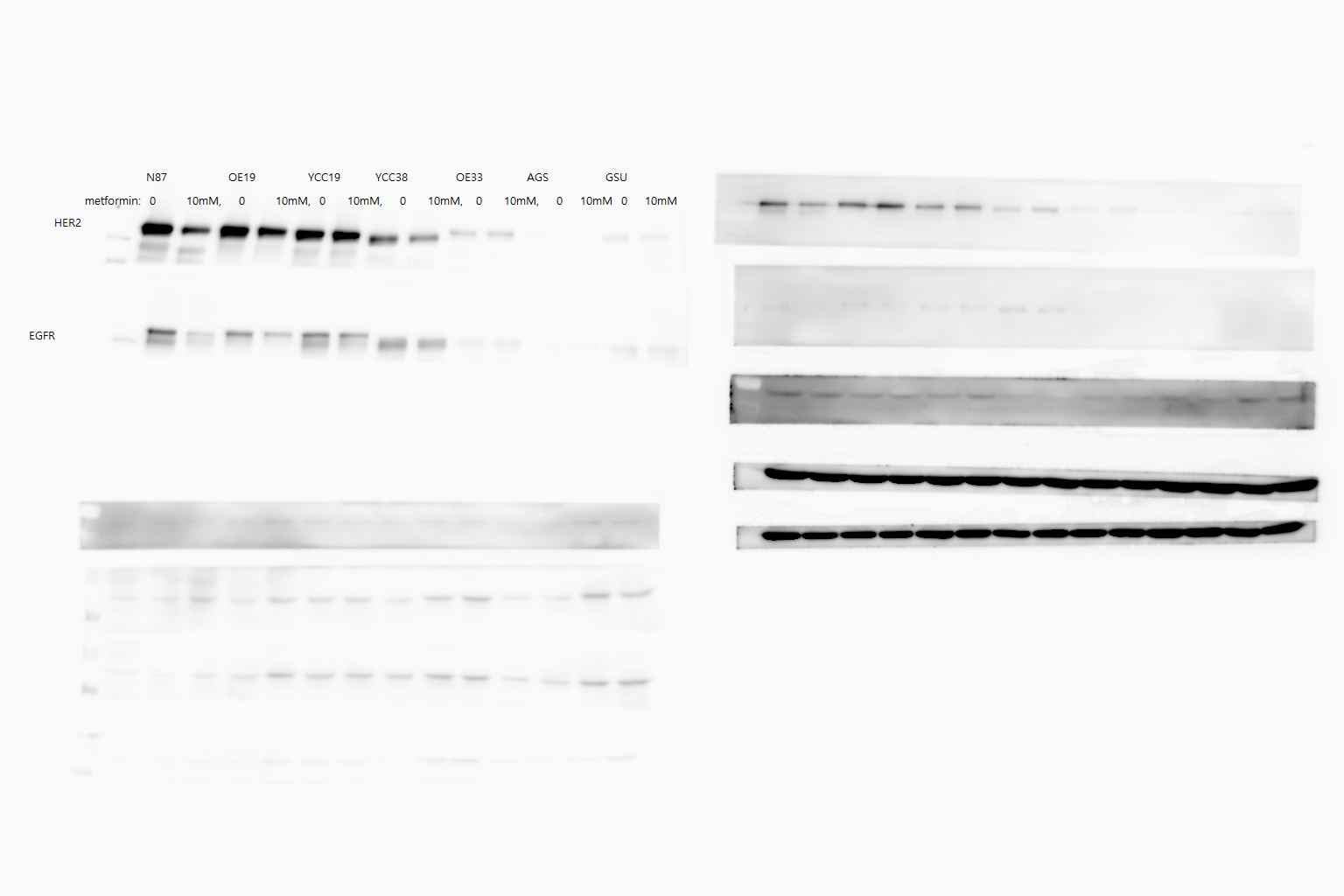

Supplement: Supplementary file 1 [file cancers-15-04768-s001.zip › cancers-2606105-File S1/Fig.2/20180606_1657_16_HER2_label.tif]

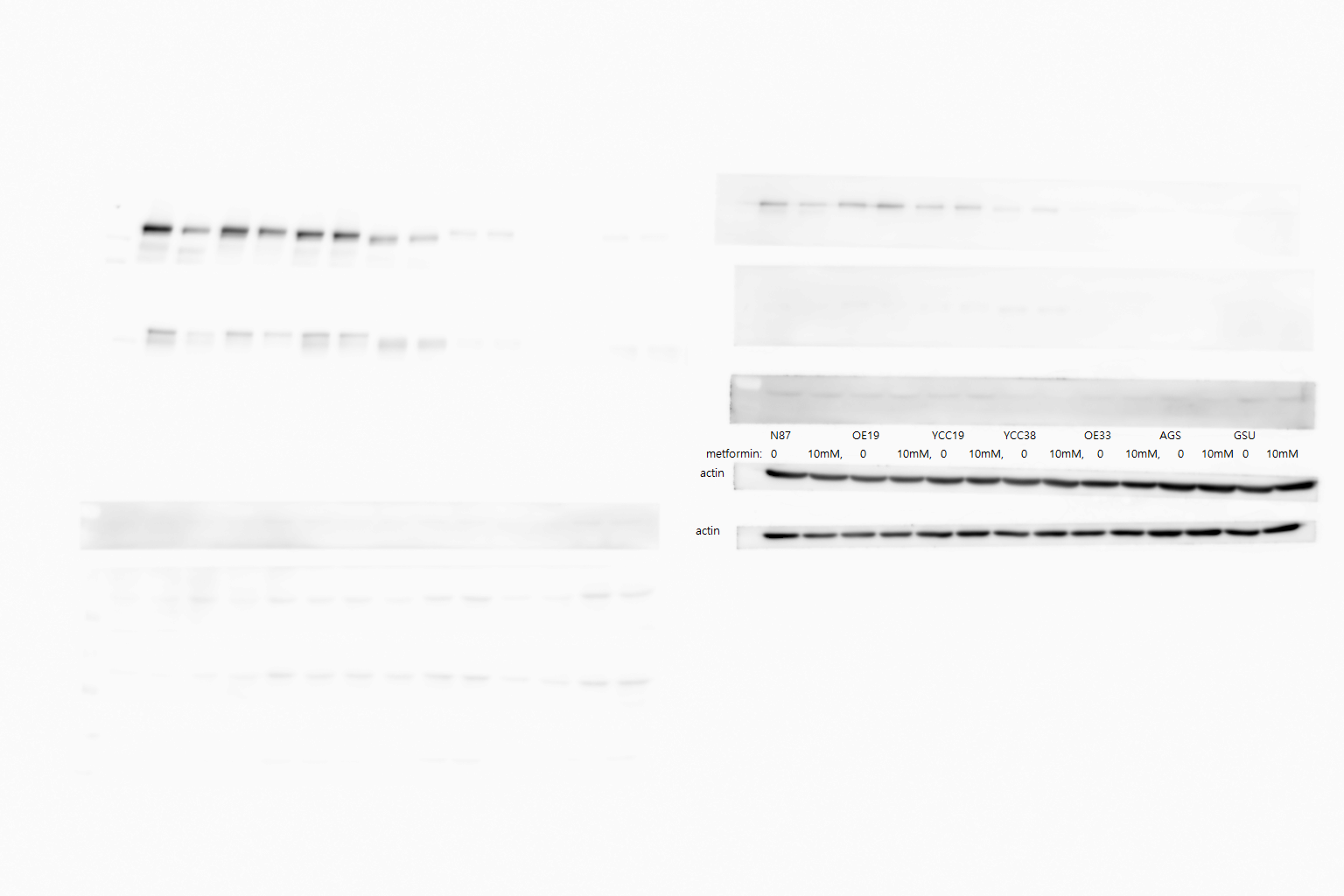

Supplement: Supplementary file 1 [file cancers-15-04768-s001.zip › cancers-2606105-File S1/Fig.2/20180606_1657_5_actin_label.tif]

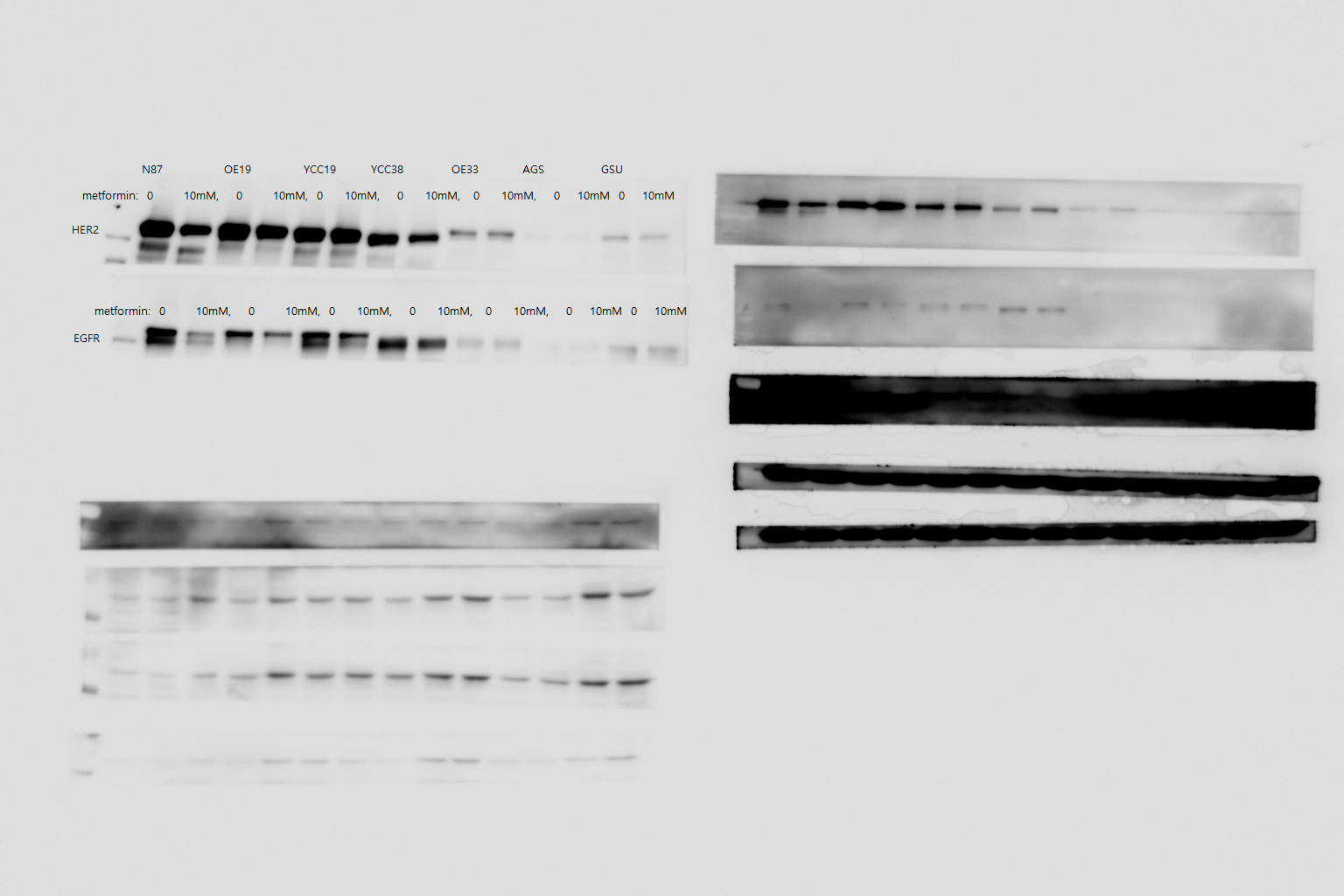

Supplement: Supplementary file 1 [file cancers-15-04768-s001.zip › cancers-2606105-File S1/Fig.2/20180606_1658_5_EGFR_label.tif]

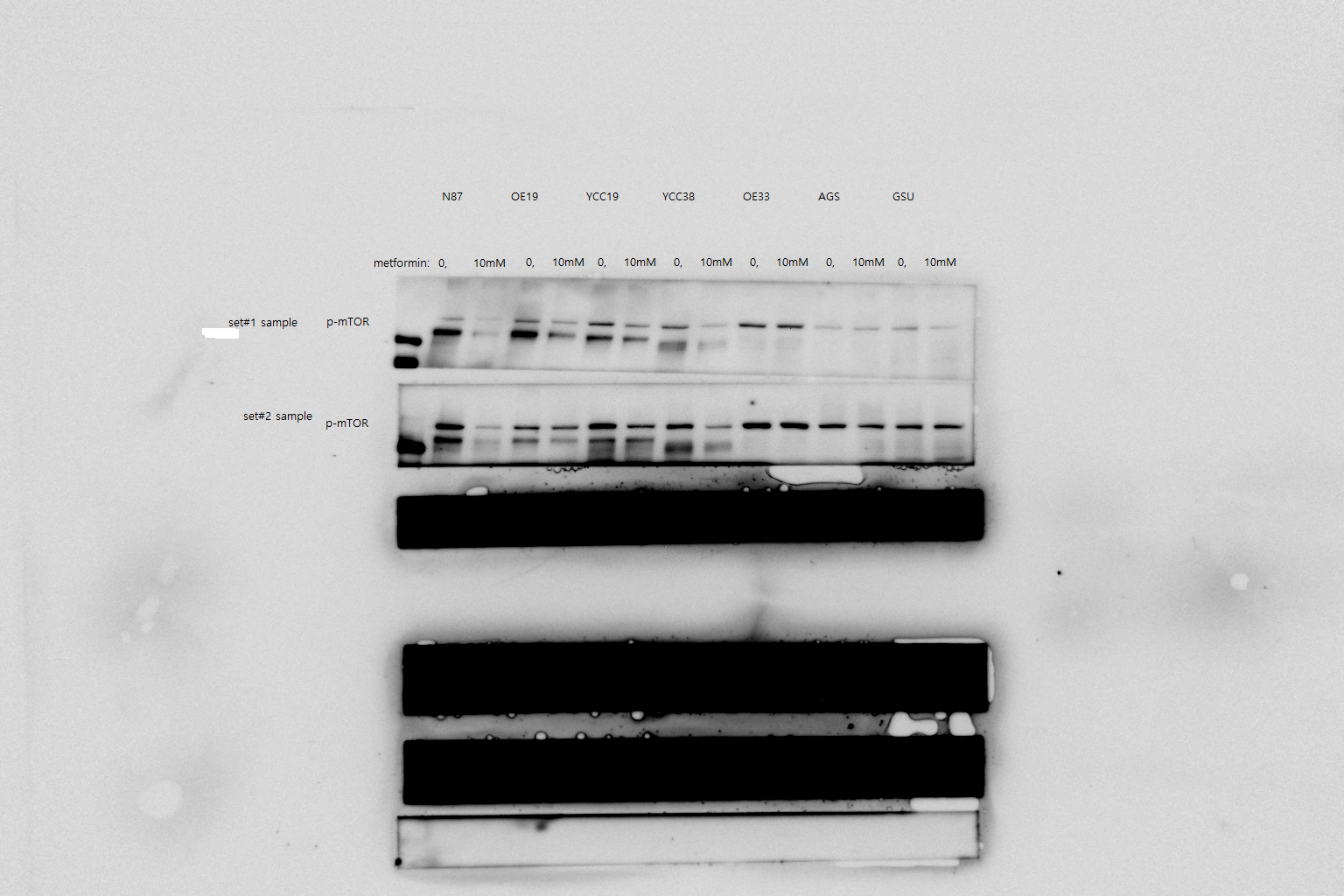

Supplement: Supplementary file 1 [file cancers-15-04768-s001.zip › cancers-2606105-File S1/Fig.2/20180608_1604_23_p-mTOR_label.tif]

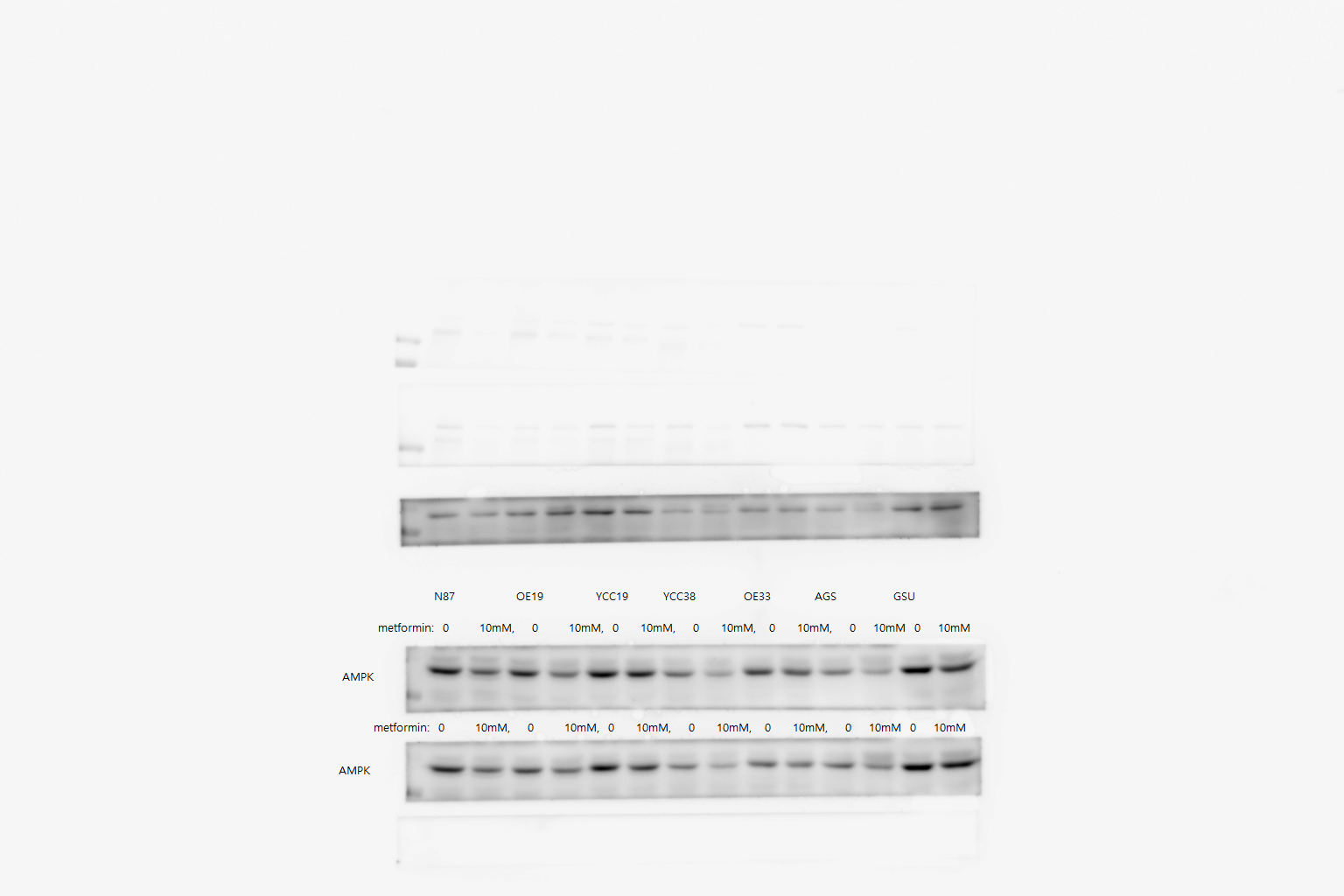

Supplement: Supplementary file 1 [file cancers-15-04768-s001.zip › cancers-2606105-File S1/Fig.2/20180608_1610_5_AMPK_label.tif]

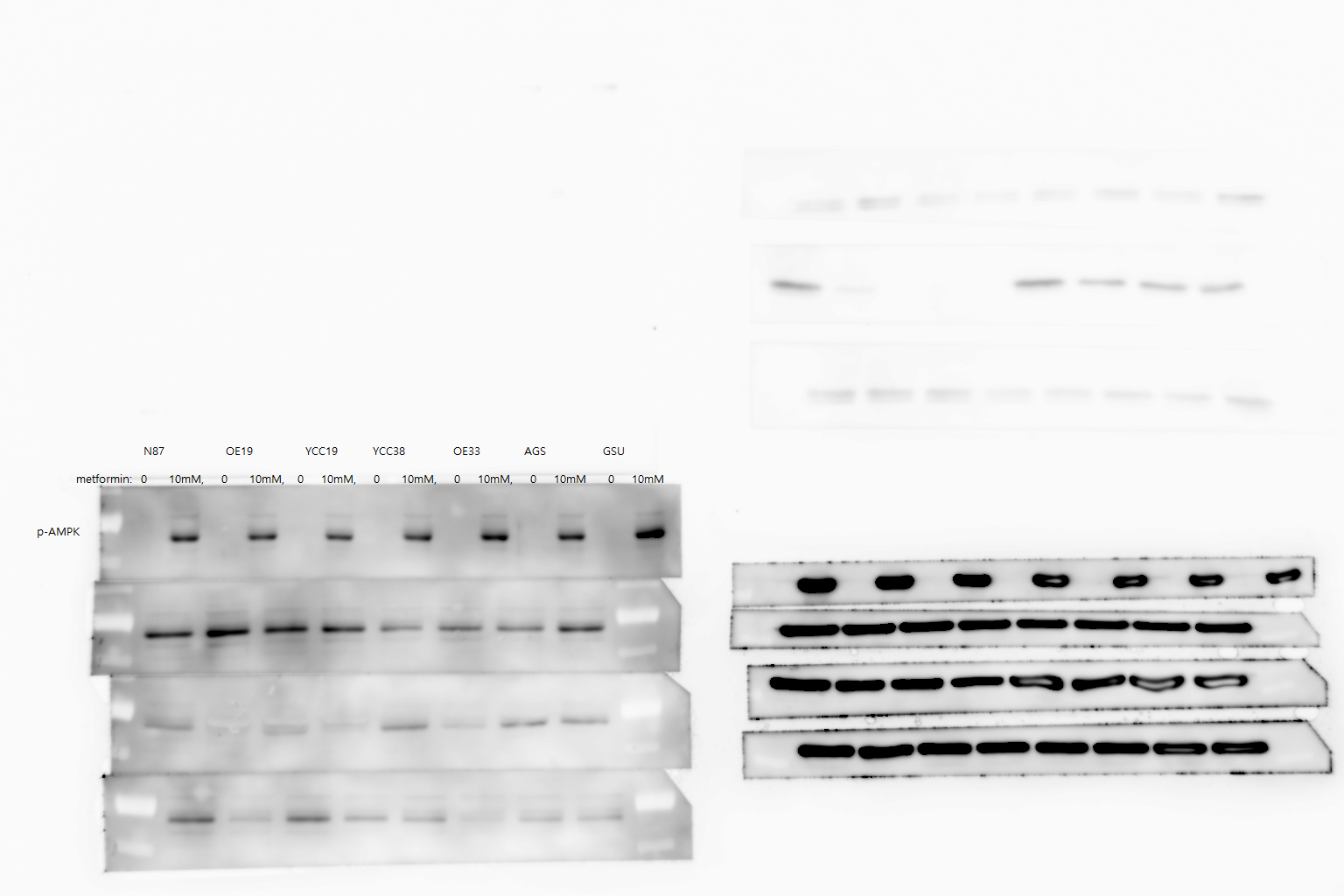

Supplement: Supplementary file 1 [file cancers-15-04768-s001.zip › cancers-2606105-File S1/Fig.2/20180619_1154_9_p-AMPK_label.tif]

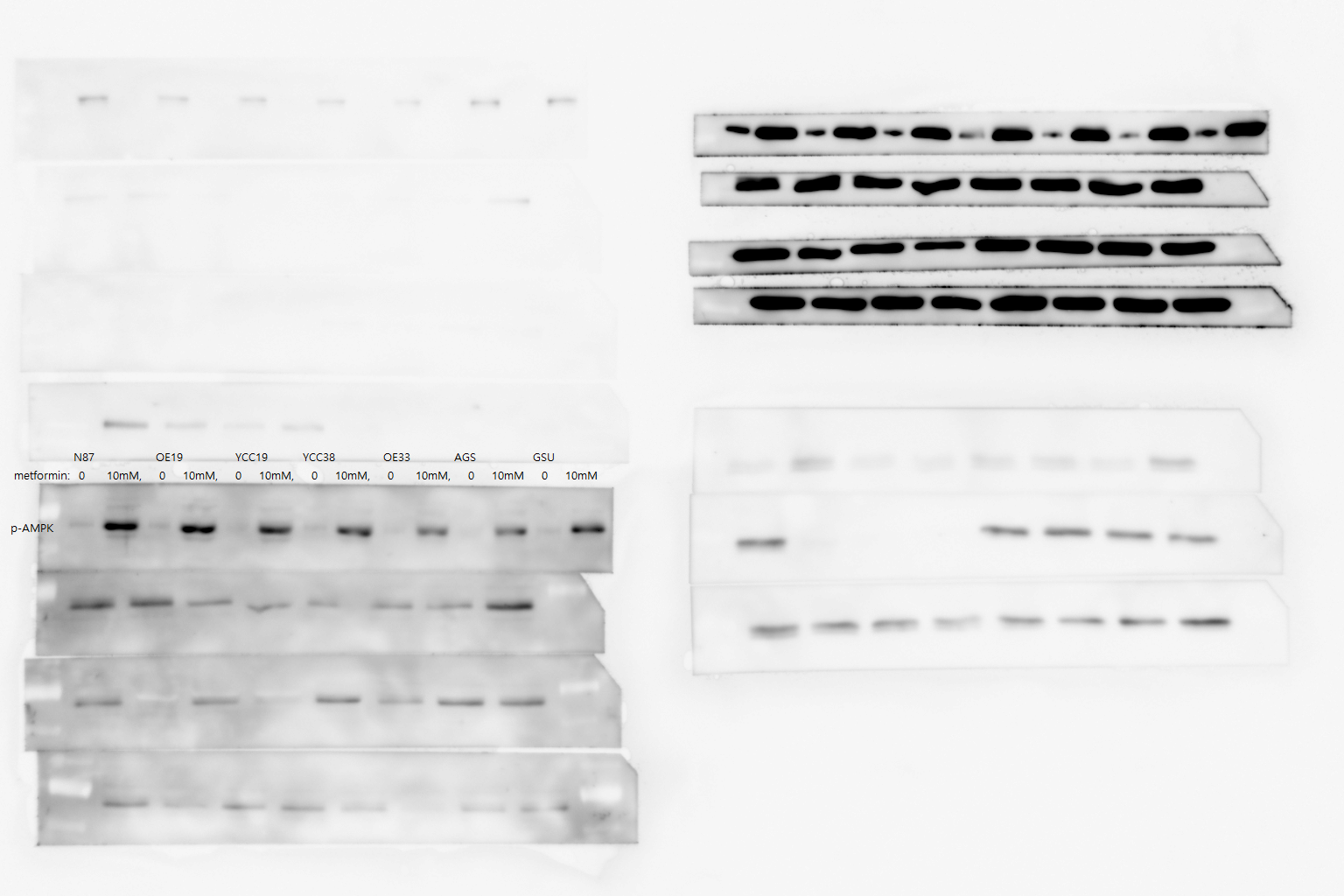

Supplement: Supplementary file 1 [file cancers-15-04768-s001.zip › cancers-2606105-File S1/Fig.2/20180620_1143_16_p-AMPK_label.tif]

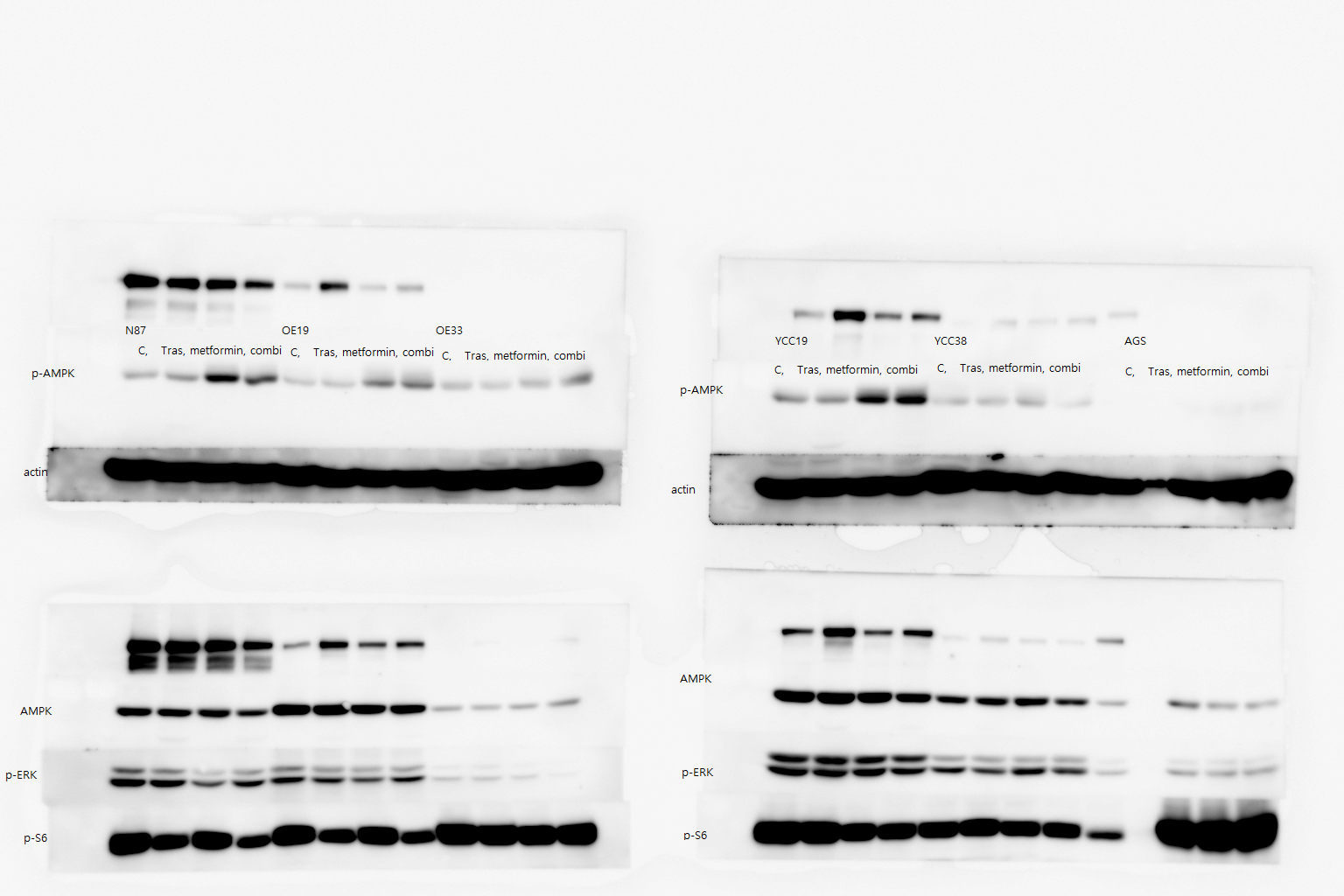

Supplement: Supplementary file 1 [file cancers-15-04768-s001.zip › cancers-2606105-File S1/Fig.4A/20170420_1534_20_pAMPK_label.tif]

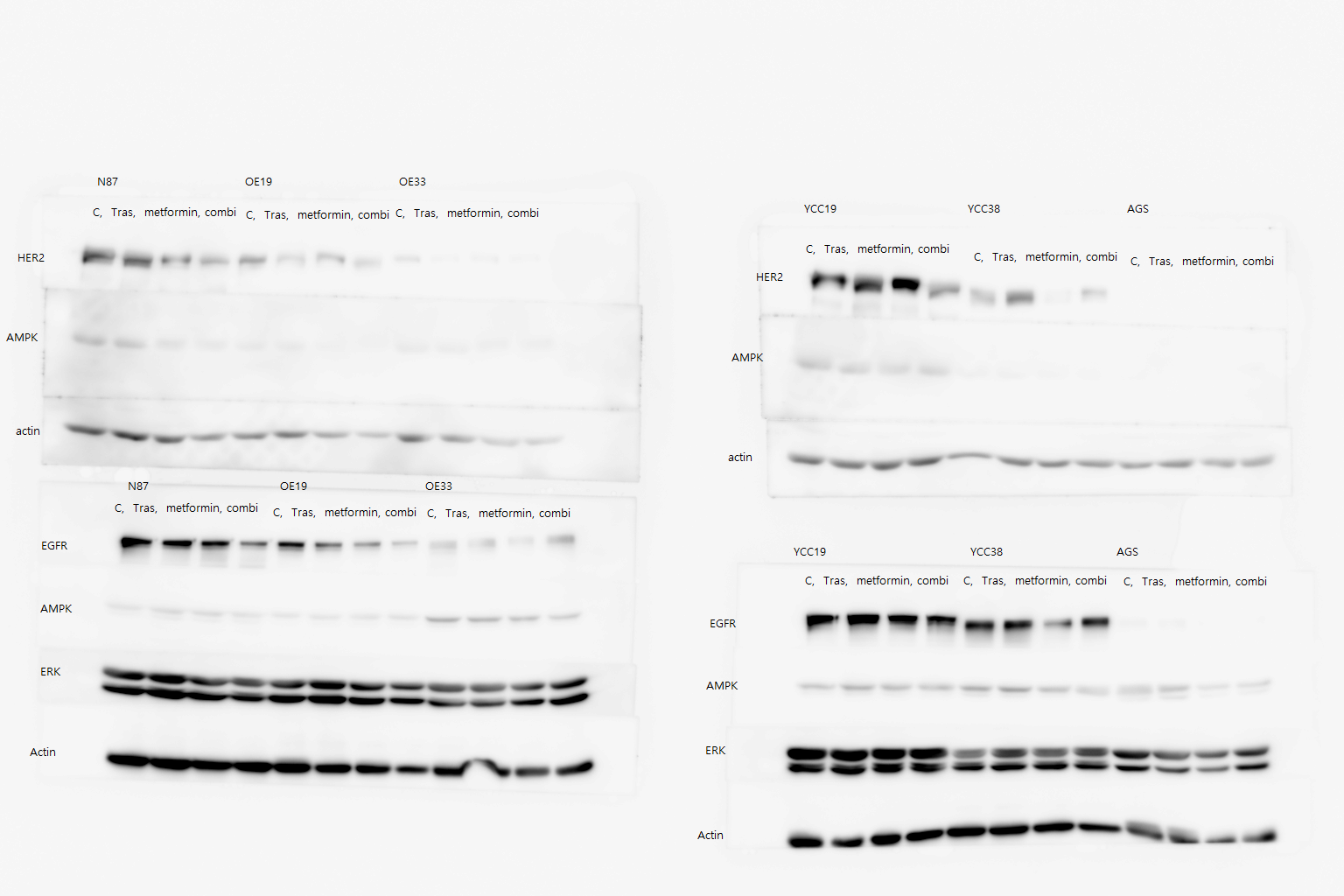

Supplement: Supplementary file 1 [file cancers-15-04768-s001.zip › cancers-2606105-File S1/Fig.4A/20170426_1701_2_total HER2_EGFR_AMPK_Actin_label.tif]

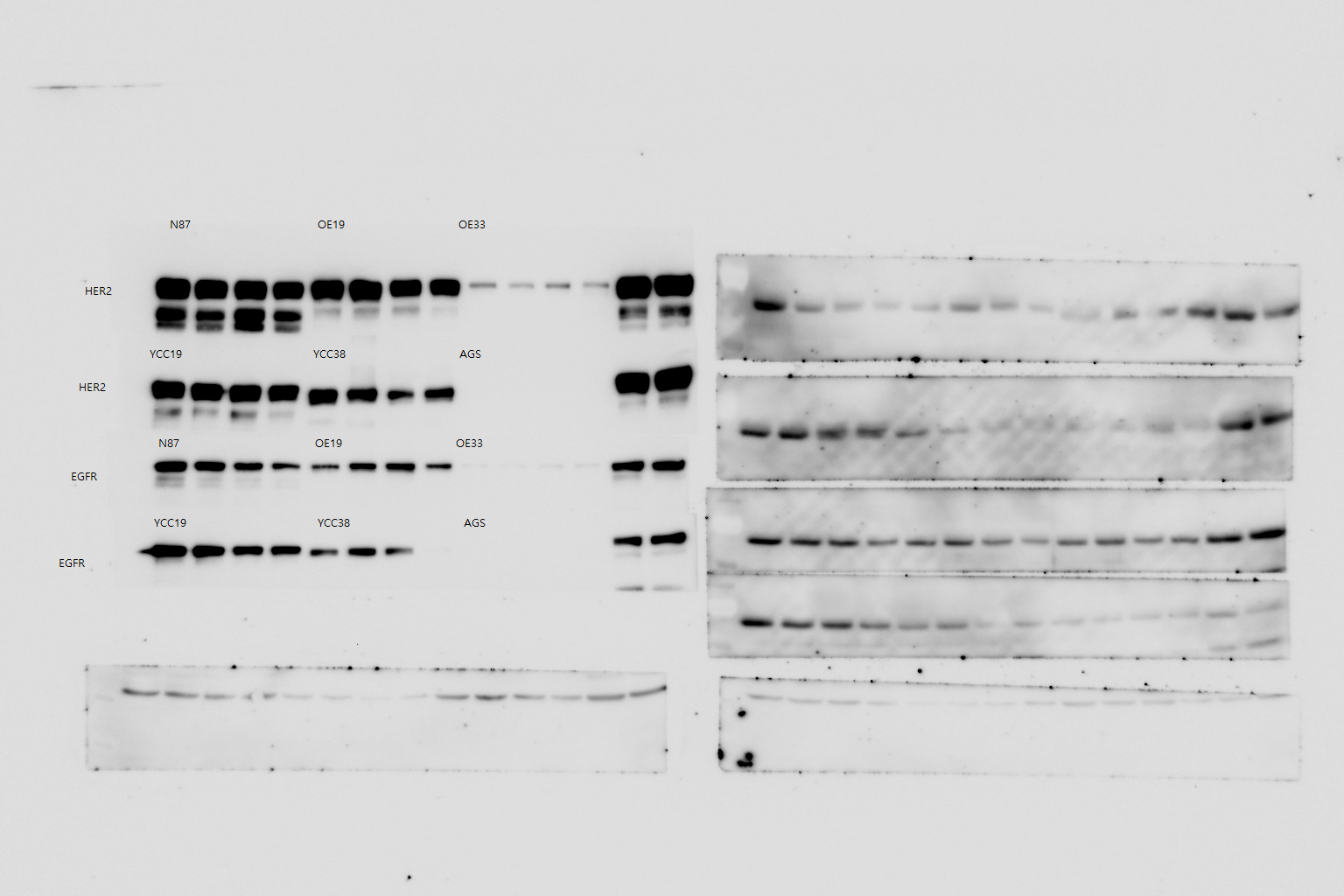

Supplement: Supplementary file 1 [file cancers-15-04768-s001.zip › cancers-2606105-File S1/Fig.4A/20170524_1132_4_HER2_EGFR_label.tif]

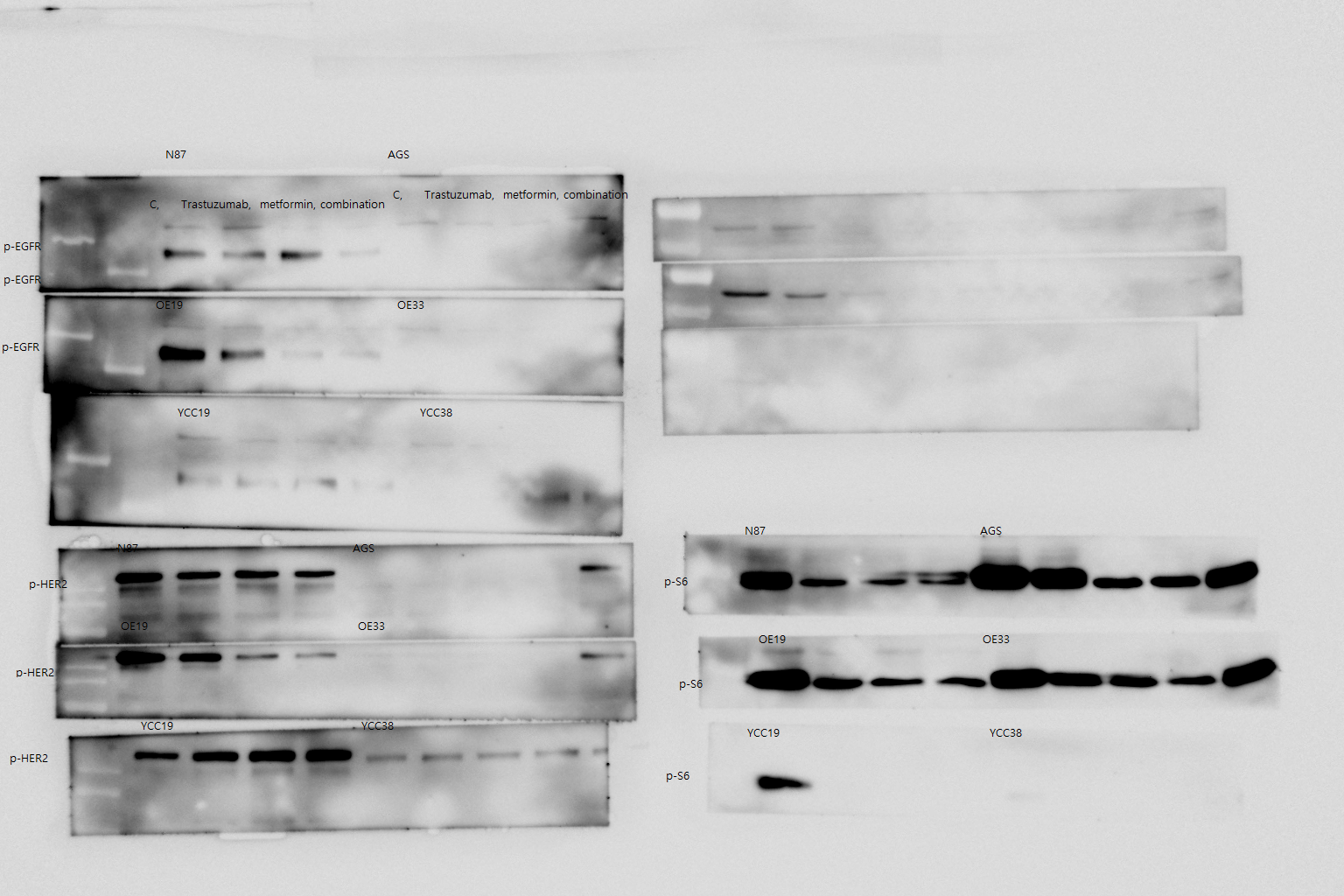

Supplement: Supplementary file 1 [file cancers-15-04768-s001.zip › cancers-2606105-File S1/Fig.4A/20171020_1922_13_p-HER2_p-EGFR_p-S6_label.tif]

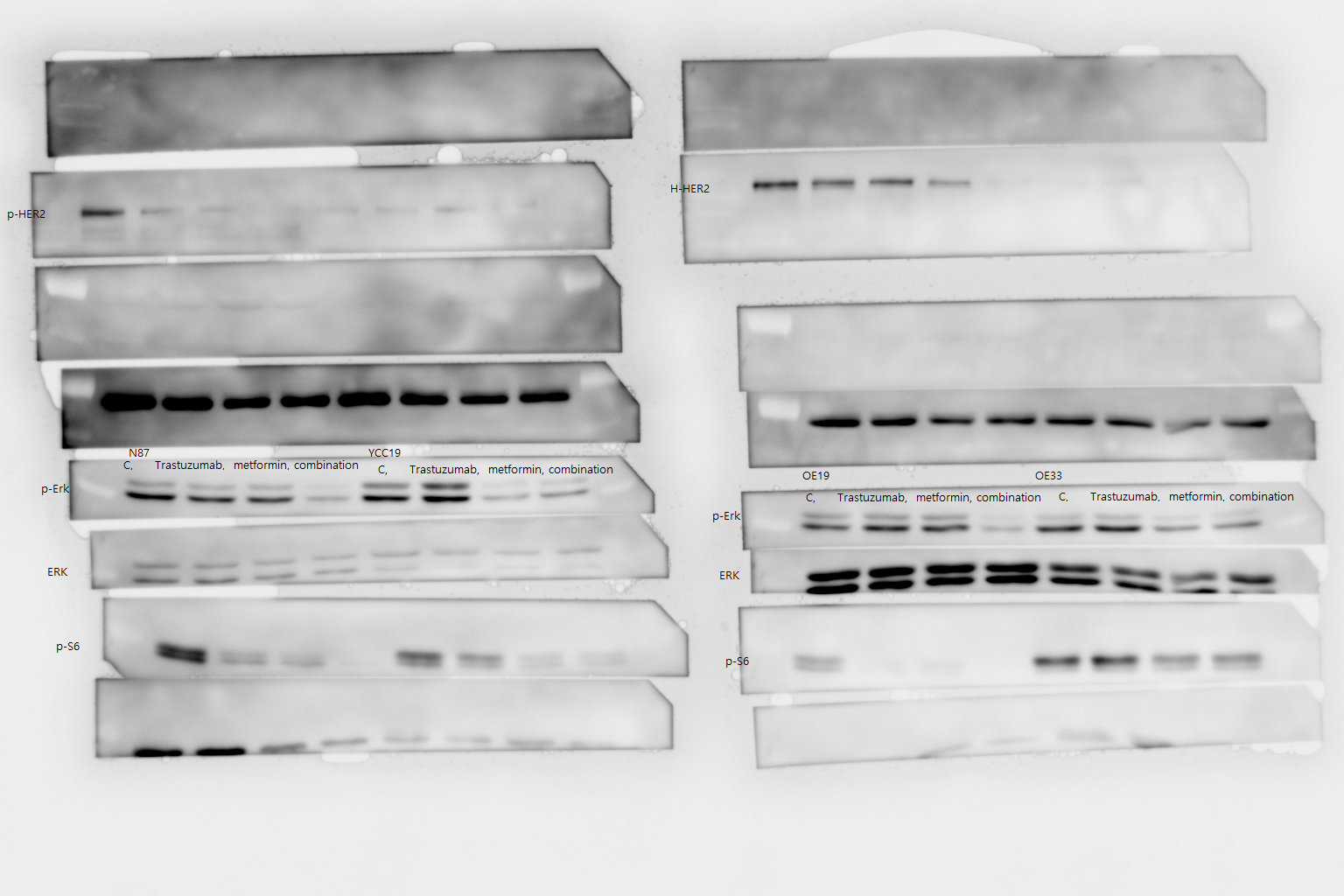

Supplement: Supplementary file 1 [file cancers-15-04768-s001.zip › cancers-2606105-File S1/Fig.4A/20180426_1649_7_p-ERK_label.tif]

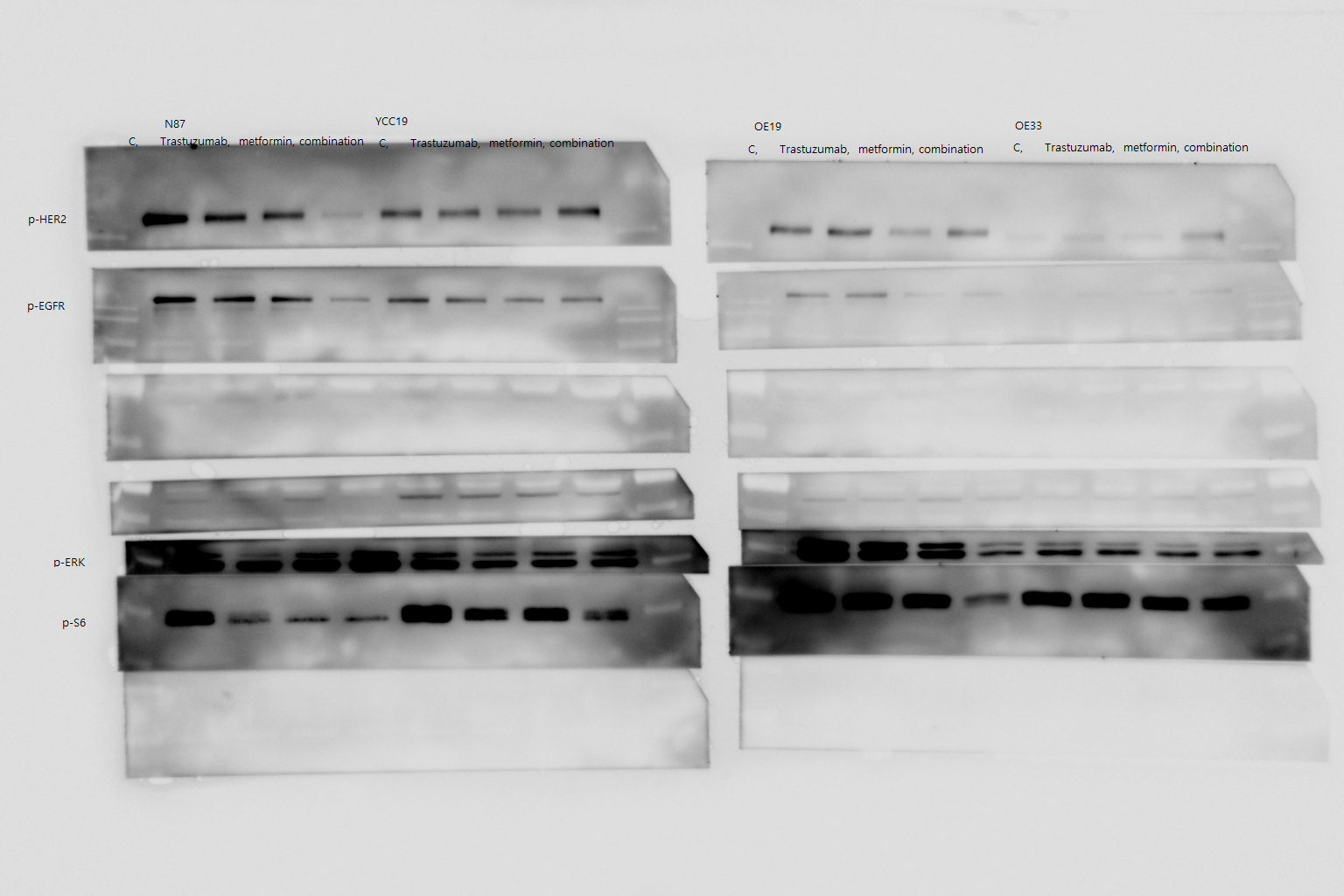

Supplement: Supplementary file 1 [file cancers-15-04768-s001.zip › cancers-2606105-File S1/Fig.4A/20180528_1622_4_p-HER2_p-EGFR_label.tif]

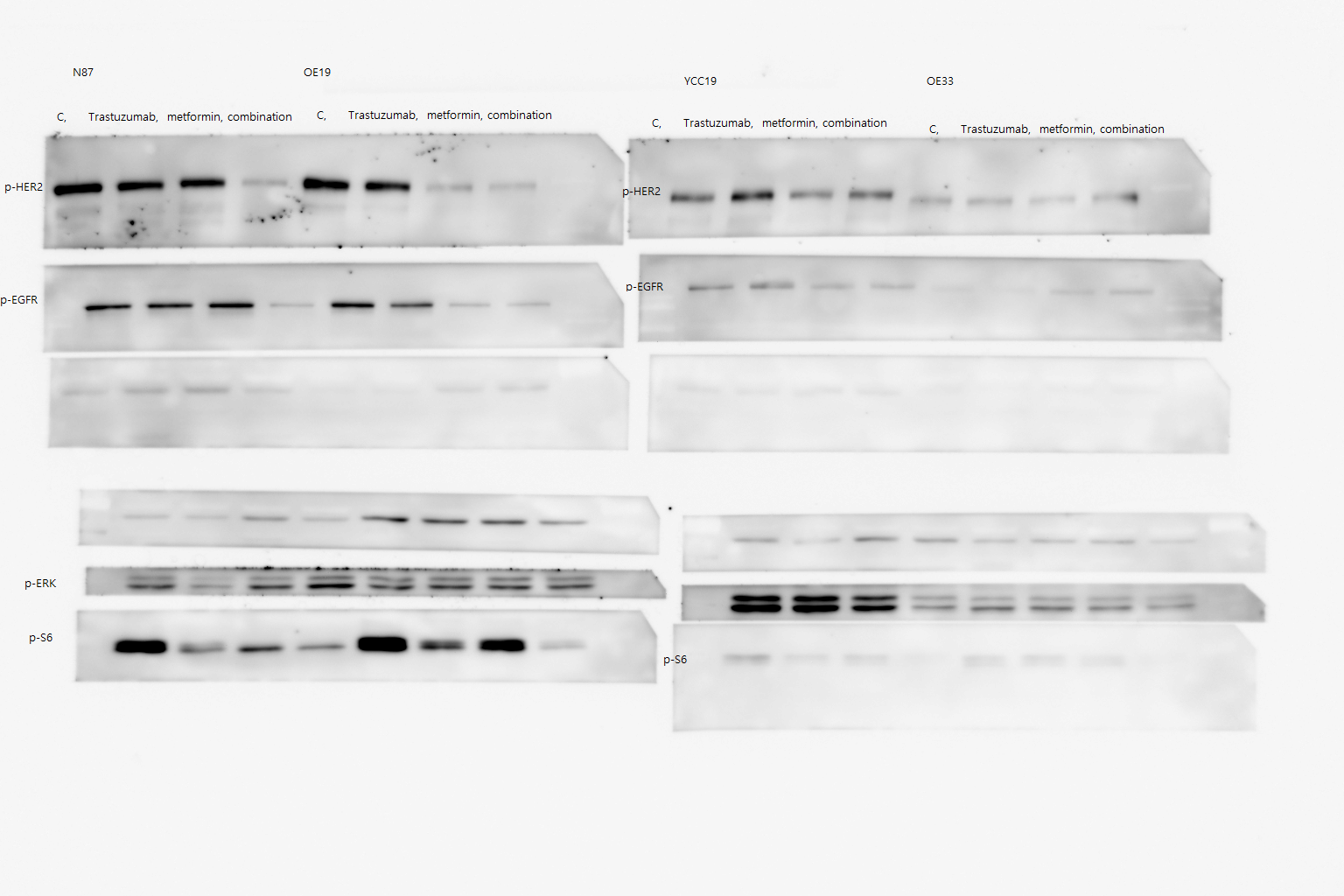

Supplement: Supplementary file 1 [file cancers-15-04768-s001.zip › cancers-2606105-File S1/Fig.4A/20180610_1350_15_p-HER2_p-EGFR_label.tif]

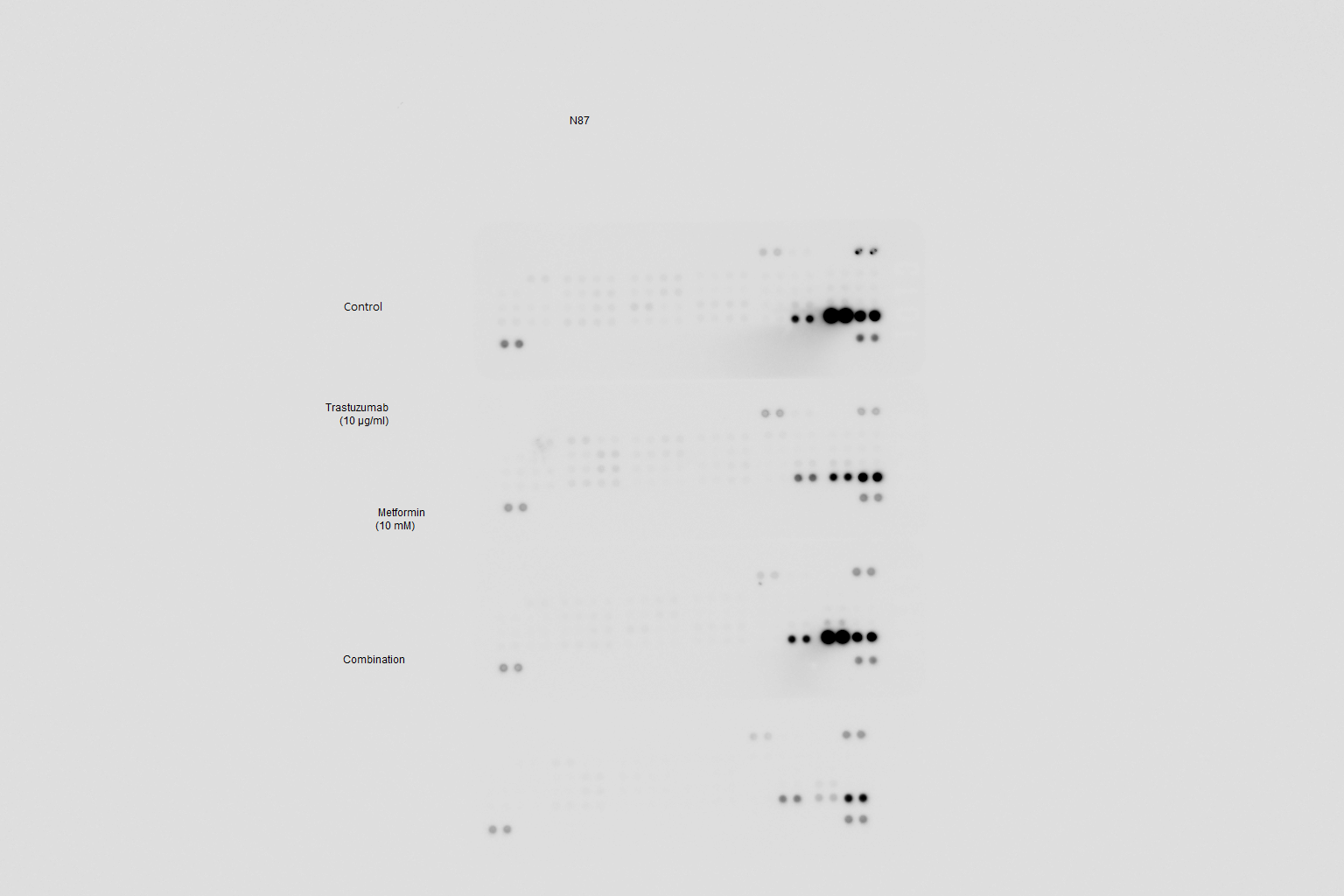

Supplement: Supplementary file 1 [file cancers-15-04768-s001.zip › cancers-2606105-File S1/Fig.4B/20180329_1541_2_N87_label.tif]

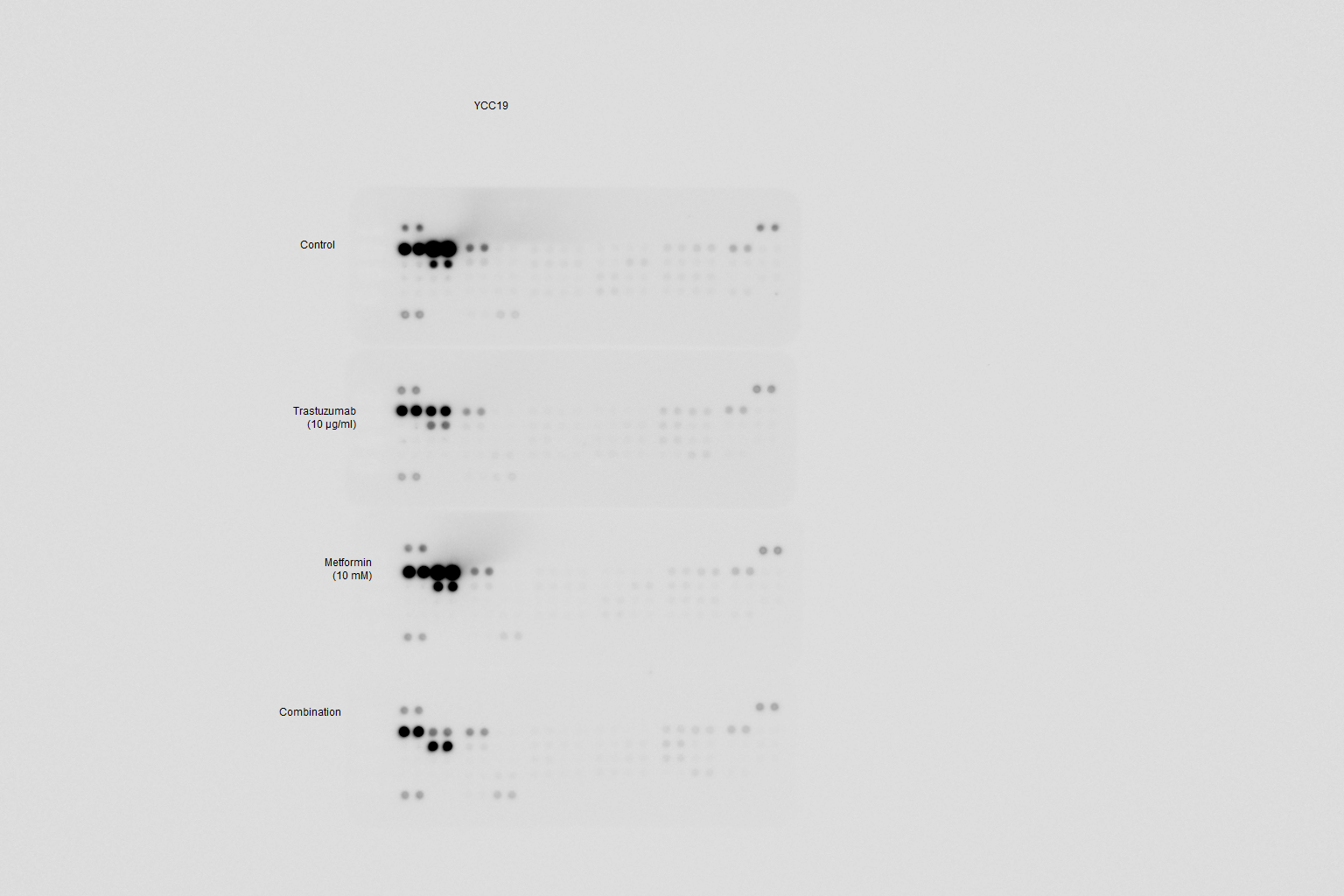

Supplement: Supplementary file 1 [file cancers-15-04768-s001.zip › cancers-2606105-File S1/Fig.4B/20180404_1447_5_YCC19_label.tif]

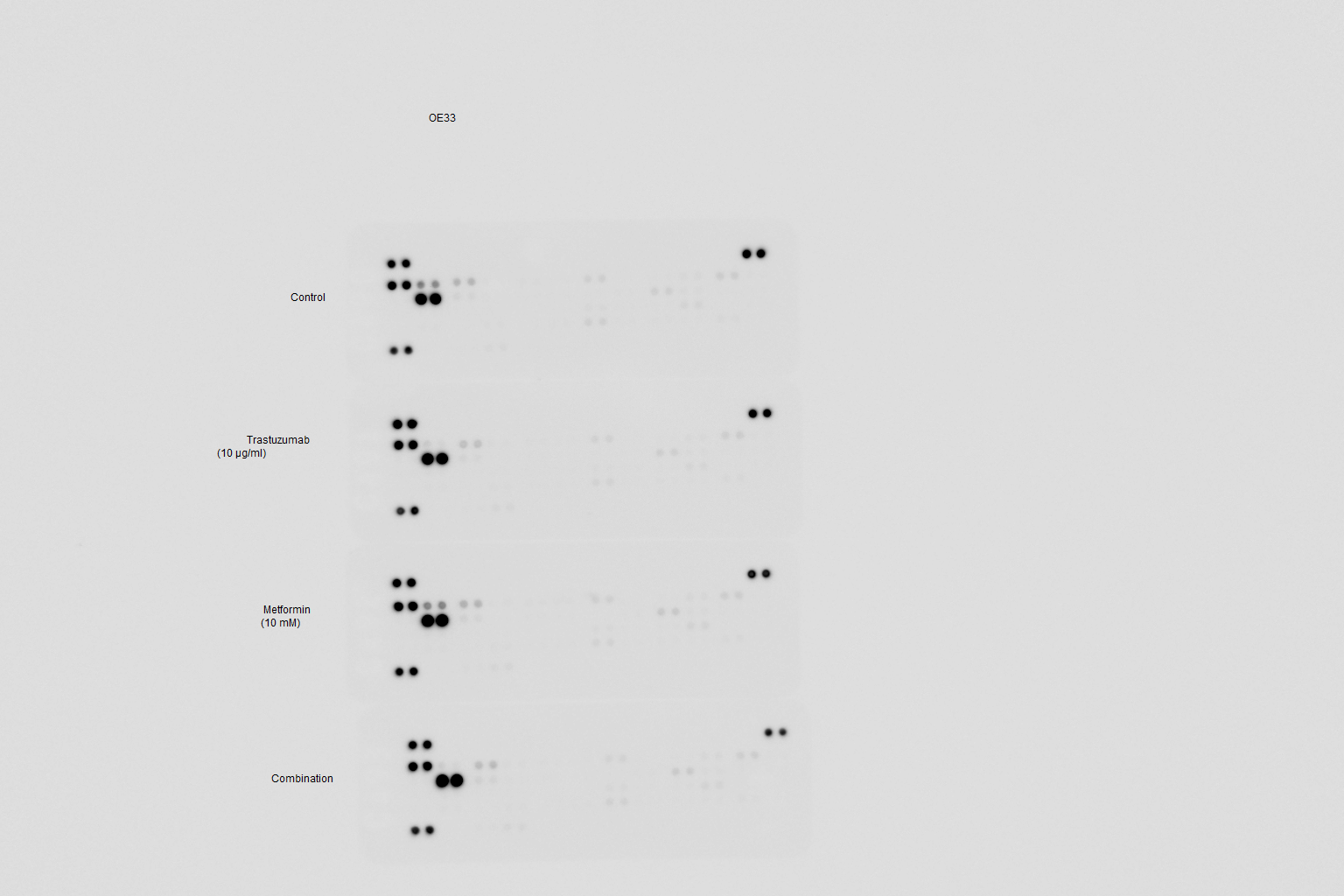

Supplement: Supplementary file 1 [file cancers-15-04768-s001.zip › cancers-2606105-File S1/Fig.4B/20180424_1426_4_OE33_label.tif]

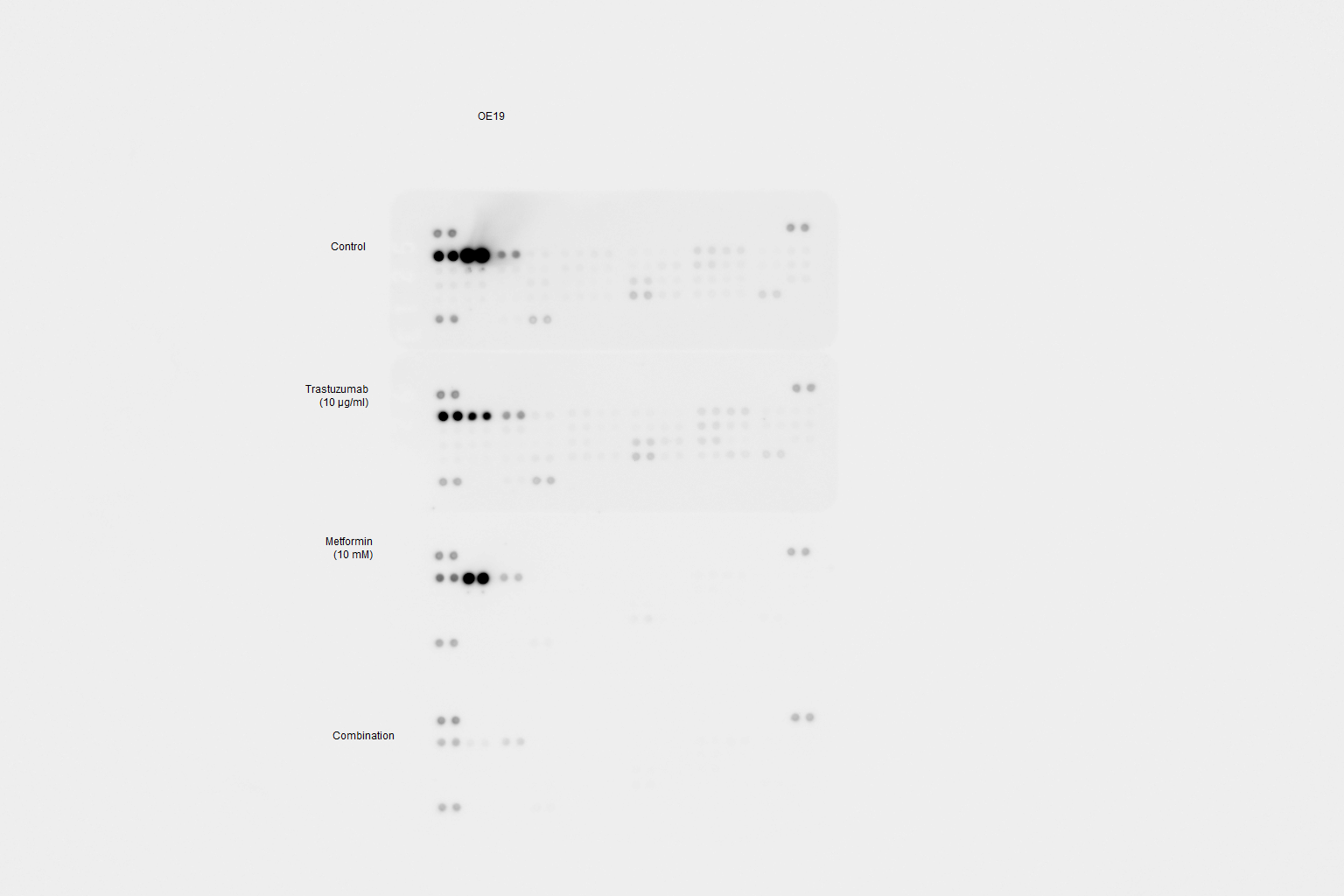

Supplement: Supplementary file 1 [file cancers-15-04768-s001.zip › cancers-2606105-File S1/Fig.4B/20180529_1450_5_OE19_label.tif]

## Slide 1
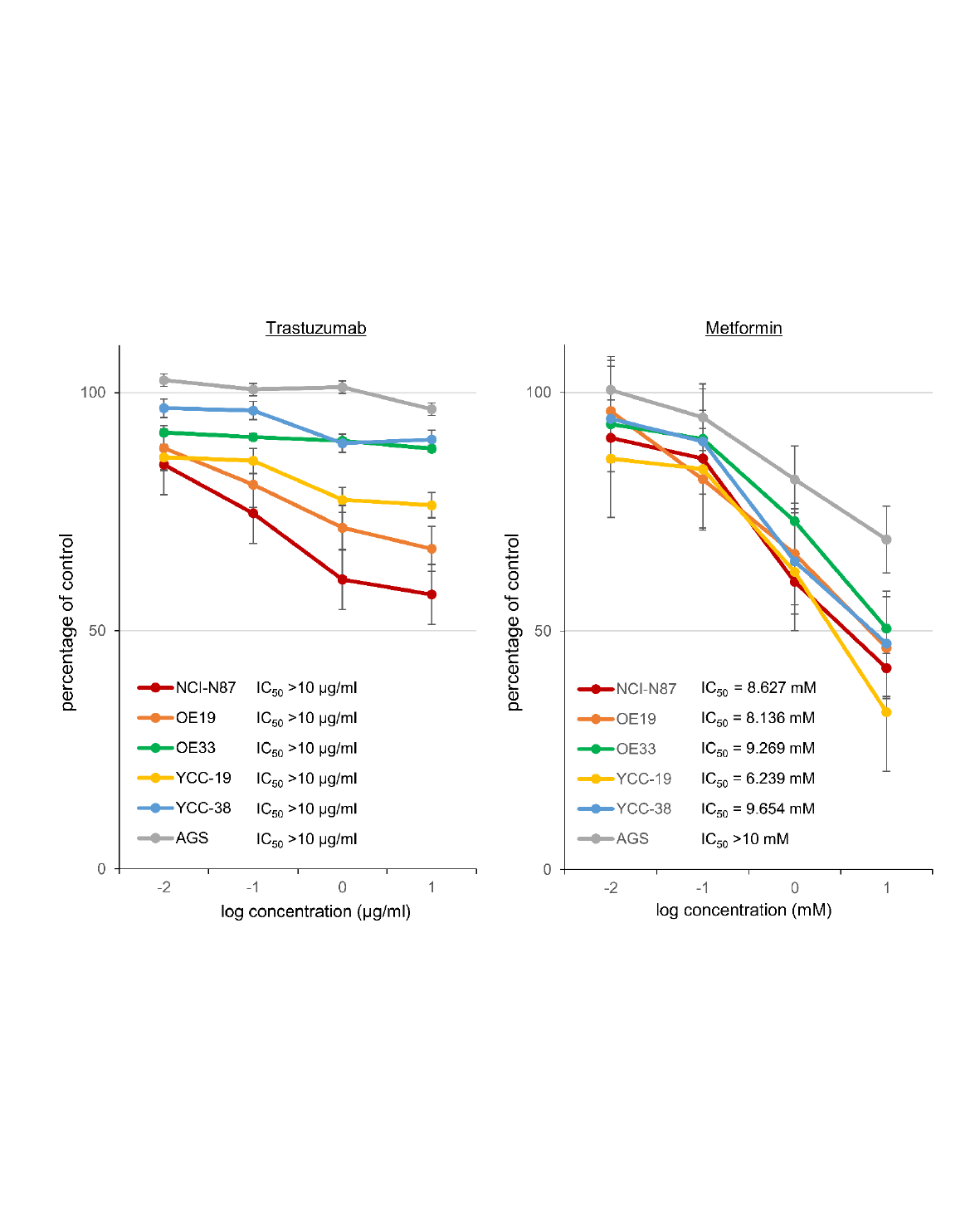

Supplement: Supplementary file 1 [file cancers-15-04768-s001.zip › Supplementary Figure S2.pptx]
